# Fungal Abundance and Diversity in the Mariana Trench, the Deepest Ecosystem on Earth

**DOI:** 10.3390/jof10010073

**Published:** 2024-01-16

**Authors:** Stefano Varrella, Giulio Barone, Cinzia Corinaldesi, Alessio Giorgetti, Hidetaka Nomaki, Takuro Nunoura, Eugenio Rastelli, Michael Tangherlini, Roberto Danovaro, Antonio Dell’Anno

**Affiliations:** 1 Department of Life and Environmental Sciences, Polytechnic University of Marche, Via Brecce Bianche, 60131 Ancona, Italy; giulio.barone89@gmail.com (G.B.); a.giorgetti@pm.univpm.it (A.G.); r.danovaro@univpm.it (R.D.); 2 National Biodiversity Future Centre, 90133 Palermo, Italy; c.corinaldesi@univpm.it; 3 Institute for Marine Biological Resources and Biotechnology, National Research Council, Largo Fiera della Pesca 2, 60125 Ancona, Italy; 4 Department of Materials, Environmental Sciences and Urban Planning, Polytechnic University of Marche, Via Brecce Bianche, 60131 Ancona, Italy; 5 X-Star, Japan Agency for Marine-Earth Science and Technology (JAMSTEC), Yokosuka 237-0061, Japan; nomakih@jamstec.go.jp; 6 Research Center for Bioscience and Nanoscience (CeBN), JAMSTEC, Yokosuka 237-0061, Japan; 7 Department of Marine Biotechnology, Stazione Zoologica “Anton Dohrn”, Fano Marine Centre, Viale Adriatico 1-N, 61032 Fano, Italy; eugenio.rastelli@szn.it; 8 Department of Research Infrastructures for Marine Biological Resources, Stazione Zoologica Anton Dohrn, Fano Marine Centre, Viale Adriatico 1-N, 61032 Fano, Italy; michael.tangherlini@szn.it

**Keywords:** Mariana Trench, deep-sea ecosystems, fungal abundance, fungal diversity, trophic conditions

## Abstract

Hadal trenches host abundant and diversified benthic prokaryotic assemblages, but information on benthic fungi is still extremely limited. We investigated the fungal abundance and diversity in the Challenger Deep (at ca. 11,000 m depth) and the slope of the Mariana Trench in comparison with three sites of the adjacent abyssal plain. Our results indicate that trench sediments are a hotspot of fungal abundance in terms of the 18S rRNA gene copy number. The fungal diversity (as the number of amplicon sequence variants, ASVs) was relatively low at all sites (10–31 ASVs) but showed a high turnover diversity among stations due to the presence of exclusive fungal taxa belonging to *Aspergillaceae*, *Trichosphaeriaceae*, and *Nectriaceae*. Fungal abundance and diversity were closely linked to sediment organic matter content and composition (i.e., phytopigments and carbohydrates), suggesting a specialization of different fungal taxa for the exploitation of available resources. Overall, these findings provide new insights into the diversity of deep-sea fungi and the potential ecological role in trench sediments and pave the way for a better understanding of their relevance in one of the most extreme ecosystems on Earth.

## 1. Introduction

The hadal zone encompasses habitats, such as trenches, at water depths exceeding 6000 m [1,2] and represents one of the most remote and least explored ecosystems on Earth [3]. The Pacific Ocean hosts the largest number of hadal trenches, including the Mariana Trench, which contains the deepest site on Earth, the Challenger Deep (at ca. 11,000 m depth [4,5]). Despite their extreme conditions (e.g., high hydrostatic pressure, complete darkness, low temperatures), hadal trenches can host unforeseen high biodiversity driven by an array of environmental factors [6,7,8,9], including the complex bottom topography and hydrodynamic. Hadal trenches, originated by the subduction of tectonic plates, show a typical V shape [10], which can influence the depositional flux of organic matter by funneling it towards the trench seafloor [11,12,13]. Thus, despite a well-documented decrease in the downward fluxes of organic matter with increasing water depth [14,15], bottom trenches can act as depocenters of organic matter, which can sustain higher microbial biomass and activity compared to adjacent abyssal plains [16,17,18,19,20,21,22,23]. Recent studies highlighted that hadal systems can host distinct prokaryotic assemblages with peculiar metabolic pathways and adaptive mechanisms [24,25,26,27].

Fungi are a ubiquitous component of deep-sea ecosystems, and they span from hypersaline anoxic basins [28,29] to cold seeps [30] and hydrothermal vents [31,32] either in surface and subsurface sediments [33,34,35,36], playing an important role in deep-sea biogeochemical processes and nutrient cycling [37,38,39,40,41]. In particular, fungi are known to contribute to the degradation of complex organic polymers [42,43] and denitrification processes [44], and their diversity is influenced by a variety of environmental factors (e.g., temperature, salinity, nutrient availability [45,46]). However, information on fungal taxonomic and functional diversity in trench systems is still scant, and factors controlling their distribution and diversity are largely unexplored [26,47,48,49].

Here, we investigated the fungal abundance (in terms of 18S rRNA gene copy number) and diversity (through the metabarcoding of the fungal internal transcribed spacer rRNA region 1) in the surface sediments of deep-sea sites characterized by different environmental conditions and ecological settings (the landward slope site and the Challenger Deep of the Mariana Trench and three sites on the adjacent abyssal plain). This study aimed to provide new insights into the relevance, diversity, and ecology of fungi in these highly remote and extreme ecosystems of the ocean.

## 2. Materials and Methods

### 2.1. Study Area and Sample Collection

**Figure 1:**
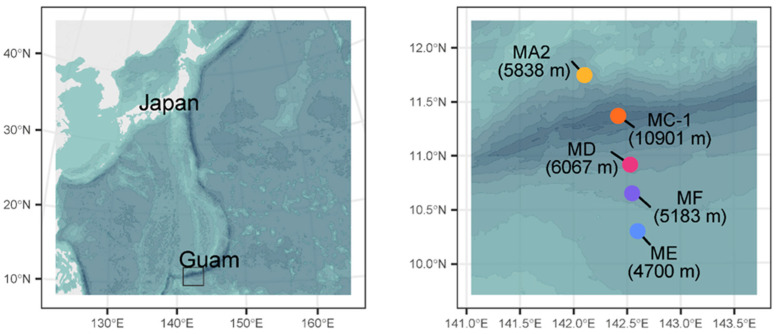
Location of the study area (**left panel**) and investigated sites across the Mariana Trench (**right panel**). Maps were generated from the GEBCO digital elevation model (https://www.gebco.net/data_and_products/gridded_bathymetry_data/, accessed on 15 October 2023).

The Mariana Trench is located in an oligotrophic area off Guam [50] along the Izu–Bonin–Mariana subduction system, extending for 2550 km and reaching a maximum depth of ca. 11,000 m at the Challenger Deep site. Surface sediment samples were collected during the KR14-01 cruise (January 2014) on board the research vessel *Kairei* using multiple-corer deployment or a lander system equipped with three sediment core samplers. Samplings were carried out at five sites located along a perpendicular transect crossing the Mariana Trench from the northern to the southern adjacent abyssal plains (Figure 1, [51]).

Site MA2 was located in the landward slope at a depth of 5838 m; sites ME and MF were located in the abyssal plains at depths of 4700 and 5183 m, respectively; site MC-1 in the Challenger Deep was located at a depth of 10,901 m and site MD in the upper part of the south slope of the Mariana Trench was located at a depth of 6067 m. Aliquots of sediment from the upper 2 cm were collected and stored at −20 °C until laboratory analyses were carried out to determine the quantity of organic matter and its biochemical composition, as well as the abundance, diversity, and taxonomic composition of fungal assemblages.

At each site, the temperature and salinity values of the bottom waters were acquired via CTD casts and summarized from previous work ([51]; Supplementary Table S1). 

### 2.2. Environmental Variables

The trophic conditions in the Mariana Trench and abyssal sites were evaluated by analyzing both the quantity and biochemical composition of organic matter [52]. Phytopigments (chlorophyll-a and phaeopigments) in surface sediments were analyzed fluorometrically after extraction (12 h at 4 °C in the dark) using 90% (*v*/*v*) acetone [53]. Total phytopigment concentrations were estimated as the sum of chlorophyll-a and phaeopigment concentrations [53]. 

The analysis of the main biochemical compounds of organic matter in deep-sea sediments (proteins, carbohydrates, and lipids) was carried out spectrophotometrically, as previously described [53]. Protein, carbohydrate, and lipid concentrations in the sediments were determined using standard curves generated with known concentrations of bovine serum albumin, glucose, and tripalmitin, respectively. The sum of protein, carbohydrate, and lipid concentrations converted into carbon equivalents (using the conversion factors of 0.49, 0.40, and 0.75 gC g^−1^, respectively) was defined as biopolymeric C content (BPC) in sediments [53].

### 2.3. Extraction and Purification of DNA for Molecular Analysis

Prior to DNA extraction, sediment samples of approximately 1 g (wet weight) were processed according to [53] to remove the presence of polymerase chain reaction inhibitors and extracellular DNA. The extraction and purification of the DNA from the sediment samples were carried out using the PowerSoil DNA isolation kit (QIAGEN, Hilden, Germany) following the standard kit protocol. The quantity and purity of the extracted DNA were checked using a Nanodrop ND 1000 (Thermo Fisher Scientific, Worcester, MA, USA). Aliquots of DNA were subsequently used for quantifying fungal 18S rRNA gene copies via real-time PCR, and fungal diversity and assemblage composition were determined via Illumina sequencing.

### 2.4. Estimates of Fungal Abundance via Quantitative Real-Time PCR (qPCR)

The DNA extracted from sediment sub-samples (*n* = 3) collected at each study site was used for quantitative real-time PCR (qPCR) analysis targeting the fungal 18S rRNA gene [54]. The 18S rRNA genes were amplified with the primer pair FR1 (5′-AICCATTCAATCGGTAIT-3′) and FF390 (5′-CGATAACGAACGAGACCT-3′), which amplify a DNA fragment of approximately 350 bp. The presence of a single PCR product of the expected length size was checked using 1% agarose gel electrophoresis stained with GelRed^®^ Nucleic Acid Gel Stain (Biotium, Landing Parkway Fremont, Fremont, CA, USA). The qPCR reactions were carried out in a volume of 15 µL according to the protocol of the Sensi-FAST SYBR Q-PCR kit (Bioline, London, UK). Each reaction contained 8 µL of Sensi-FAST master mix, primers at a final concentration of 1 μM, and 1 μL of DNA template [54]. The amplification procedure was as follows: 94 °C for 3 min; 40 cycles (denaturation at 94 °C for 10 s, annealing at 50 °C for 15 s, and extension at 72 °C for 20 s) on a thermal cycler Bio-Rad iQ^™^5 (Bio-Rad, Hercules, CA, USA). All reactions were carried out in triplicates. Different concentrations of the known 18S rRNA gene copies of *Aspergillus niger* were used for preparing qPCR standard curves. Sample concentrations were determined using the iQ^™^5 Optical System software (version 2.1) after checking the efficiency and R^2^ of each standard curve. To exclude potential qPCR biases due to the presence of inhibitors, reactions were run using undiluted aliquots of isolated DNA, in addition to running all sample extracts in serial 10-fold dilutions. The log-linear relationship between the cycle threshold (Ct) and dilution factor was observed in all samples using a 10-fold dilution. The quantity of 18S rRNA gene copies was reported per gram of sediment dry weight.

### 2.5. Fungal Diversity and Assemblage Composition

For the analysis of fungal diversity, the nuclear ribosomal internal transcribed spacer 1 (ITS1) was amplified using the primer set ITS1F (5′-GGAAGTAAAAGTCGTAACAAGG-3′; and ITS2 (5′-GCTGCGTTCTTCATCGATGC-3′) [55,56]. The sequencing was performed by the LGC group (Berlin, Germany) on the Illumina MiSeq platform. Raw sequences were then processed using QIIME^™^ 2 version 2023.2 [57]. Adapters and primer fragments were removed with the command q2-cutadapt [58]. The ITSxpress plugin version 1.8 was then employed to trim sequences that target the ITS1 region [59]. Trimmed paired-end sequences were denoised, error-corrected in marginal sequences, and joined, and chimeric and singleton sequences were removed via the DADA2 procedure with default parameters [60]. The Amplicon Sequence Variant (ASV) table was subsequently rarefied to 728 randomly selected sequences, which correspond to the lowest read count observed in our samples [61]. The resulting ASVs were taxonomically identified using the UNITE database (version: 9.0; last updated: 17 October 2022) [62]. The ITSx tool version 1.1 used on the PlutoF webserver (https://plutof.ut.ee/, accessed on 22 September 2023) [63,64] was applied to the representative sequences obtained by DADA2 to further remove non-ITS1 sequences and thus improve the quality of the dataset. Taxonomic assignment was performed on the refined ASV set via the SINTAX tool in Usearch v11 using a threshold value of 0.8 [65,66]. Alpha diversity, based on the Shannon index, was calculated with the diversity plugin using the R package vegan (version 2.6-4; [67]).

### 2.6. Data Analysis

To test for differences in trophic variables indicated by biochemical analyses and fungal abundance (as 18S rRNA gene copy number) among sites, analysis of variance (one-way ANOVA) was carried out. The normality of variables was evaluated with Shapiro’s test, and variance homogeneity was evaluated with Levene’s test. When significant differences were encountered, pair-wise comparisons using Bonferroni correction were performed. 

To identify the main factors driving the distribution of fungal abundance, a generalized linear model (GLM) using Poisson regression analysis [68,69] was carried out using the temperature and salinity of the bottom water and variables of trophic conditions as covariates (Supplementary Table S1). Exploratory data analysis was previously carried out to reduce potential multicollinearity among covariates using both Pearson’s correlation coefficients [69] and variance inflation factors (VIF; [70,71]). Covariates were rescaled using a min–max normalization, and those showing a correlation with values greater than 0.75 were excluded [72]. From these analyses, only total phytopigment and carbohydrate concentrations were selected for investigating the role of such covariates in explaining the distribution pattern of the fungal pattern. Then, model selection was performed to identify the best model, minimizing the Akaike information criterion value (AIC) [73,74] and discarding all models with no significant coefficients or those that violate the assumption of independence of the response observations [74]. All visualizations were generated in RStudio Team (2020), whereas statistical analyses were performed in Python 3.9 via pandas and statsmodels packages [75,76]. The network plot for visualizing shared and exclusive ASVs among sampling sites was generated with Gephi 0.10.1 [77].

Finally, a redundancy analysis (RDA; [78]) based on Euclidean distances has been carried out to investigate relationships between fungal assemblage composition (at the family level) and the same set of covariates identified by the previous multicollinearity detection analysis using the R package *microeco* (v1.3.0) [79]. 

## 3. Results and Discussion

**Figure 2:**
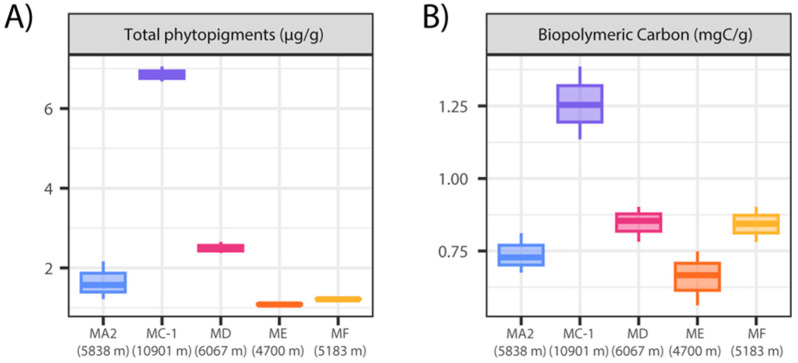
Boxplots of the concentrations of total phytopigments (**A**) and biopolymeric carbon (**B**) in the sediments collected at different benthic deep-sea sites located in the abyssal plains (MA2, ME, and MF sites), south upper slope (site MD), and Challenger Deep (site MC-1) of the Mariana Trench. Colors correspond to the site’s name reported on the x-axis.

Deep-sea ecosystems depend on the export flux of primary production from the ocean’s surface waters, but organic matter fluxes typically decrease exponentially with increasing water depth, thus potentially limiting the biological assemblages at abyssal and hadal depths [14,15,19]. The Mariana Trench and the surrounding abyssal plain are located near a subtropical gyre characterized by very low photosynthetic production [80], which is expected to provide a limited export of organic matter to deep-sea sediments. The results of the present study confirmed this expectation as the phytopigment concentrations in the abyssal sediments adjacent to the Mariana Trench (sites MA2, ME and MF; Figure 2A) were much lower than the values observed at comparable depths in more productive oceanic areas (e.g., NE Atlantic Ocean) [52,81]. However, the fresh organic matter concentrations (expressed as phytopigments’ content) in the surface sediments of the Mariana Trench at ca. 11,000 m depth (site MC-1) were 4–6 folds higher than in the adjacent abyssal or slope sites (Figure 2A; Supplementary Table S2A,B). The Challenger Deep sediments, accordingly, were characterized by relatively high concentrations of biopolymeric C (i.e., a proxy of trophic resource availability [52]; Figure 2B; Supplementary Tables S1 and S2A,B), which reflect the ability of trenches to channel organic material towards the deepest portion, favored by the V-shape trench morphology [11,12,13,22]. 

**Figure 3:**
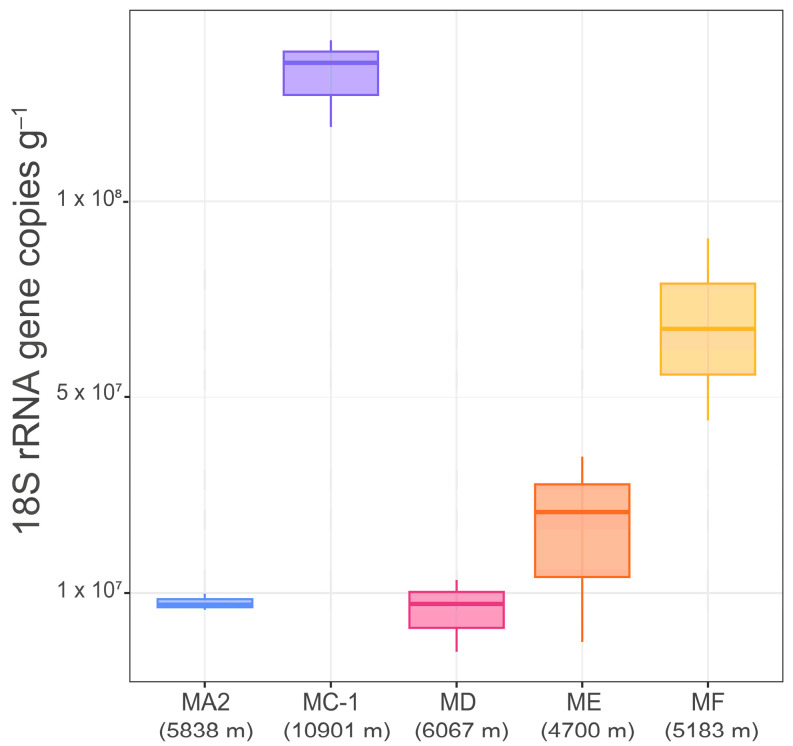
Boxplot of fungal abundance (expressed as the number of 18S rRNA gene copies per gram of dry sediment) in the sediments collected at different benthic deep-sea sites located in the abyssal plains (MA2, ME, and MF sites), south upper slope (site MD), and Challenger Deep (site MC-1) of the Mariana trench. Colors correspond to the site name reported on the x-axis.

There is a wide consensus that food availability (expressed as organic matter concentration) is one of the main factors controlling the abundance and biodiversity of deep-sea benthic assemblages, from prokaryotes to meio- and macrofauna [19,82,83]. Here, we show that the bottom of the Mariana Trench, which behaves as a depocenter of organic matter, is a hot spot of fungal abundance (expressed as fungal 18S rRNA gene copies per gram of dry sediment). The Challenger Deep site showed fungal abundance (on average 2.13 ± 0.53 × 10^8^ 18S rRNA gene copies g^−1^) that is much higher than the one of the slope and abyssal sediments (ranging from 0.09 ± 0.02 to 0.52 ± 0.27 × 10^8^ at MD and MF sites, respectively; Figure 3; Supplementary Table S3A,B). The fungal abundances in the Challenger Deep are also among the highest values reported so far in the world’s deep-sea sediments (all data based on qPCR; [31,34,66,84]). 

Using a generalized linear model, we found a significant relationship between the abundance of benthic fungi and the concentrations of carbohydrates and total phytopigments (Supplementary Tables S4 and S5), with the latter alone explaining 79% of the total fungal variance. Overall, these findings indicate that the organic matter enrichment observed in Mariana Trench sediments can sustain large abundances of fungi, as previously observed for prokaryotic assemblages [21,51]. Such a high fungal abundance could contribute to the high benthic oxygen consumption rates documented at ca. 11,000 m depth [17,20], although so far this component has not been taken into consideration. 

**Figure 4:**
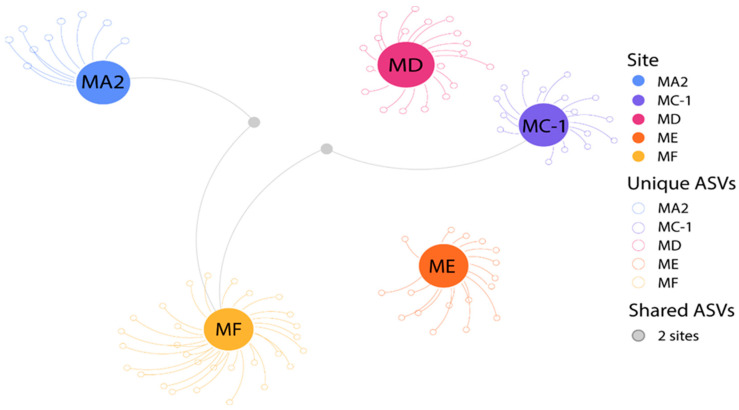
Network plot based on the assemblage composition of fungal ASVs determined in the different sediment samples collected in the five benthic deep-sea sites. Colored nodes represent sampling sites, while gray nodes represent the ASVs shared between two samples that are connected by gray edges.

In the present study, we report a total of 149,710 reads from ITS1 DNA sequencing (ranging from 2307 to 83,227 at the MA2 and MF sites, respectively), which allowed the identification of a total of 92 fungal ASVs after stringent denoising and filtering. Rarefaction curves highlighted that the sequencing depth was enough to adequately describe the fungal diversity of the deep-sea sediments examined (Figure S1). Our results indicate that the fungal ASV richness, as well as the Shannon diversity index, was relatively low when compared to the richness of fungal taxa reported from other bathyal, abyssal, and hadal sediments [34,49,66,85]. Despite the different environmental conditions (i.e., temperature, hydrostatic pressure, and food availability) of the abyssal and hadal sites investigated, fungal ASV richness varied within a narrow range (from 10 to 31 fungal ASVs at the MA2 and MF sites, respectively; Table 1). However, most fungal ASVs were uniquely reported in a single site (Figure 4), indicating a high turnover (β-)diversity among the investigated sites. These results suggest that each of the investigated sites hosts exclusive fungal taxa, leading to distinct assemblages but contributing to a larger diversity at increasing spatial scales. Similarly, major differences have been previously reported in benthic prokaryotic diversity among abyssal and hadal sites, including the Mariana Trench [51].

**Figure 5:**
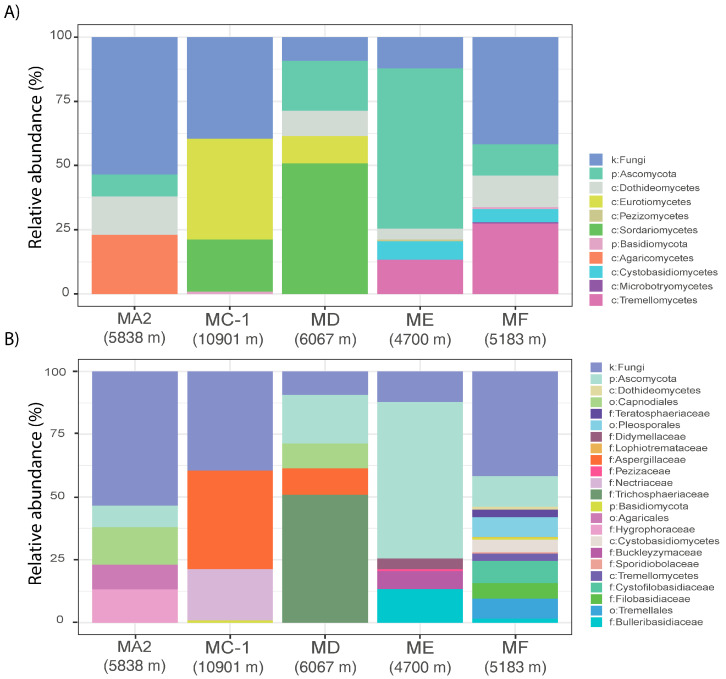
Stacked bar plot of the relative abundance of fungal taxa classified, whenever possible, down to class (**A**) and family (**B**) levels in the sediment samples collected from the five benthic deep-sea sites. Letters refer to fungal ASVs classified at kingdom (k), phylum (p), class (c), order (o), and family (f) levels.

Our dataset revealed that among the 92 taxa encountered, 29 fungal ASVs were affiliated with the phylum Ascomycota and 22 were affiliated with Basidiomycota, whereas 41 ASVs were classified as fungi at the kingdom level, which showed no matches with current fungal taxonomy at the phylum level. The relative proportion of such unclassified fungi was high at all sites (40% of the total reads at the Challenger Deep site, 54% at MA2, and 42% in the MF abyssal sites; Figure 5A). These results suggest that trench sediments and the adjacent abyssal plain can harbor novel and phylogenetically diverse fungal taxa, which can open new perspectives in the taxonomic and functional characterization of deep-sea fungal assemblages. 

Our study also highlights major differences in fungal assemblages among the investigated sites at the levels of class and family (Figure 5A,B). The Challenger Deep (site MC-1) was dominated by the phylum Ascomycota, particularly the classes Eurotiomycetes and Sordariomycetes (accounting for 39.3% and 20.3% of the total reads, respectively; Figure 5A) and families *Aspergillaceae* and *Nectriaceae* (Figure 5B). The phylum Ascomycota also dominated the fungal assemblages in the seaward slope site of the Mariana Trench (site MD), where the dominant classes were Sordariomycetes (50.8%), followed by Eurotiomycetes (10.6%) and Dothideomycetes (9.9%). The analysis conducted at the genus level did not provide sufficient information, as most sequences were unclassified (>73%), with some samples (e.g., MA-2) resulting in no classification of ASVs at such taxonomic levels. Previous studies carried out in the Izu–Ogasawara and Yap [44,49,86] and Mariana Trench sediments [87], as well as in the water column overlying the Challenger Deep [48,88], reported the presence of the phylum Ascomycota but without providing evidence of its quantitative relevance. Unknown Ascomycota taxa were extremely abundant at the ME site (62.4%), which was also characterized by taxa affiliated with the phylum Basidiomycota and belonging to the classes Tremellomycetes (13.3%) and Cystobasidiomycetes (7.1%). The fungal taxa of the phylum Basidiomycota represented an important fraction of the assemblage at the MA2 and MF sites, where Agaricomycetes and Tremellomycetes were the main classes (accounting for 23% and 27% of the total reads, respectively).

**Figure 6:**
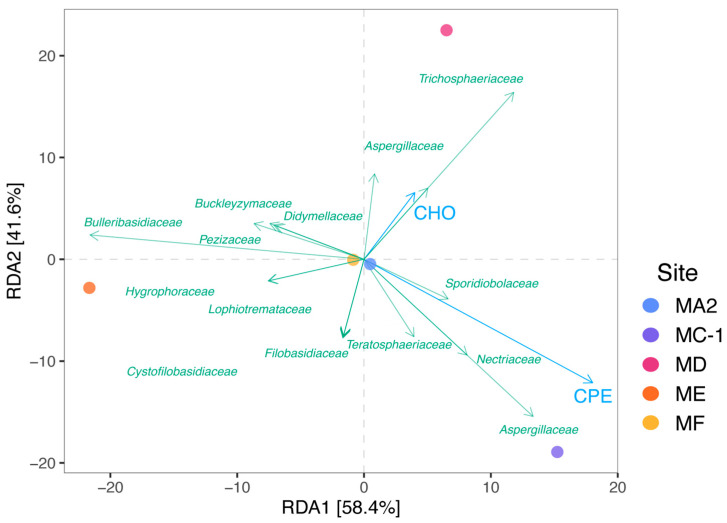
Redundancy analysis (RDA) ordination plot of the relationships between fungal assemblage composition at the family level and environmental variables determined in the sediment samples collected in the five benthic deep-sea sites. Colored dots represent benthic sites and light blue vectors represent environmental variables (CPE and CHO). CPE = total photopigment concentrations; CHO = carbohydrate concentrations.

To provide insights into potential factors influencing fungal assemblage composition, we carried out a redundancy analysis (RDA) using environmental variables as predictors and fungal taxonomic composition at the family level as the response variable (Figure 6). This analysis allowed us to identify significant positive relationships between the fungal families *Aspergillaceae* and *Trichosphaeriaceae* and carbohydrate concentrations in the sediment of the slope of the Mariana Trench, as well as between fungal families *Aspergillaceae* and *Nectriaceae* and total phytopigment concentrations in the Challenger Deep sediments. Such relationships suggest a link between these fungal taxa and the availability of specific organic sources. *Aspergillaceae* and *Nectriaceae* have been reported to be involved in the degradation of algal polysaccharides (by specific carbohydrate-active enzymes; [26,43,89,90]), which showed high concentrations in the Challenger Deep. Previous studies reported that fungal taxa affiliated with *Aspergillaceae* might preferentially utilize a refractory fraction of organic polymers (e.g., carbohydrates; [26,91]), which showed high concentrations coupled with high abundances of *Aspergillaceae* in the slope sediments investigated in the present study.

Overall, our findings indicate that the Challenger Deep is a hotspot of fungal abundance, characterized by the presence of different taxa than those of the slope of the Mariana Trench and the adjacent abyssal plains. Our results provide new insights into factors driving the abundance and diversity of deep-sea fungal assemblages and pave the way for a better understanding of their ecological role in one of the most extreme ecosystems on Earth.

## Figures and Tables

**Table 1:** Fungal richness, expressed as the number of fungal ASVs, and Shannon diversity index in the sediments of the benthic deep-sea sites investigated.

Sample Site	Depth (m)	Fungal ASVs	Shannon Diversity Index
MA2	5838	10	1.82
MC-1	10,901	17	2.05
MD	6067	19	1.84
ME	4700	17	1.96
MF	5183	31	2.83

## Data Availability

Raw sequencing reads have been deposited in the NCBI Sequence Read Archive (SRA) under accession number PRJNA1035066.

## Supplementary Materials

The following supporting information can be downloaded at https://www.mdpi.com/article/10.3390/jof10010073/s1. The Supplementary Materials include the following: Figure S1: Rarefaction curves of fungal ASVs obtained from the analysis of metabarcoding of ITS sequences: amplified from sediment samples collected in the different benthic deep-sea sites investigated; Table S1: Temperature and salinity values of the bottom waters and phytopigment, protein, carbohydrate, lipid, and biopolymeric C concentrations in the surface sediments of different deep-sea sites investigated; Table S2: Outputs of the main ANOVA test (A) and pairwise comparison test (B) carried out on the different trophic variables determined in different benthic deep-sea sites investigated; Table S3: Outputs of the main ANOVA test (A) and pairwise comparison test (B) carried out on fungal abundances (expressed as 18S rRNA gene copies g^−1^) determined in different benthic deep-sea sites investigated; Table S4: Variance inflation factor (VIF) for each covariate in the set of predictors selected; Table S5: Output of the best Poisson regression, resulting from a model selection, carried out using environmental variables (CPE and CHO) as predictors and 18S rRNA gene copies g^−1^ as response. CPE = total phytopigment concentrations; CHO = carbohydrate concentrations.
