# Supplementary material for: Fungal Abundance and Diversity in the Mariana Trench, the Deepest Ecosystem on Earth
Source: J Fungi (Basel). 2024 Jan 16;10(1):73. doi: 10.3390/jof10010073 (PMC10820024; doi:10.3390/jof10010073)
Supplement: Supplementary file 1 [file jof-10-00073-s001.zip › jof-2758295-supplementary.pdf]

Supplementary Materials

Fungal Abundance and Diversity in the Mariana Trench, the Deepest Ecosystem on Earth

Stefano Varrella[1,2]*, Giulio Barone[1,3], Cinzia Corinaldesi[2,4], Alessio Giorgetti[1], Hidetaka Nomaki[5], Takuro Nunoura[6], Eugenio Rastelli[7], Michael Tangherlini[8], Roberto Danovaro[1,2], Antonio Dell'Anno[1,2*]

[1] Department of Life and Environmental Sciences, Polytechnic University of Marche, Via Brecce Bianche, Ancona, 60131, Italy; giulio.barone89@gmail.com (G.B.); a.giorgetti@pm.univpm.it (A.G.); r.danovaro@univpm.it (R.D.)

[2] National Biodiversity Future Centre, Palermo, 90133, Italy; c.corinaldesi@univpm.it

[3] Institute for Marine Biological Resources and Biotechnology, National Research Council, Largo Fiera della Pesca 2, Ancona, 60125, Italy

[4] Department of Materials, Environmental Sciences and Urban Planning, Polytechnic University of Marche, Via Brecce Bianche, Ancona, 60131, Italy

[5] X-Star, Japan Agency for Marine-Earth Science and Technology (JAMSTEC), Yokosuka, 237-0061, Japan; nomakih@jamstec.go.jp

[6] Research Center for Bioscience and Nanoscience (CeBN), JAMSTEC, Yokosuka, 237–0061, Japan; takuron@jamstec.go.jp

[7] Department of Marine Biotechnology, Stazione Zoologica "Anton Dohrn", Fano Marine Centre, Viale Adriatico 1-N, Fano, 61032, Italy; eugenio.rastelli@szn.it

[8] Department of Research Infrastructures for Marine Biological Resources, Stazione Zoologica Anton Dohrn, Fano Marine Centre, Viale Adriatico 1-N, Fano, 61032, Italy; michael.tangherlini@szn.it

* Correspondence: s.varrella@univpm.it (S.V.); a.dellanno@univpm.it (A.D.)

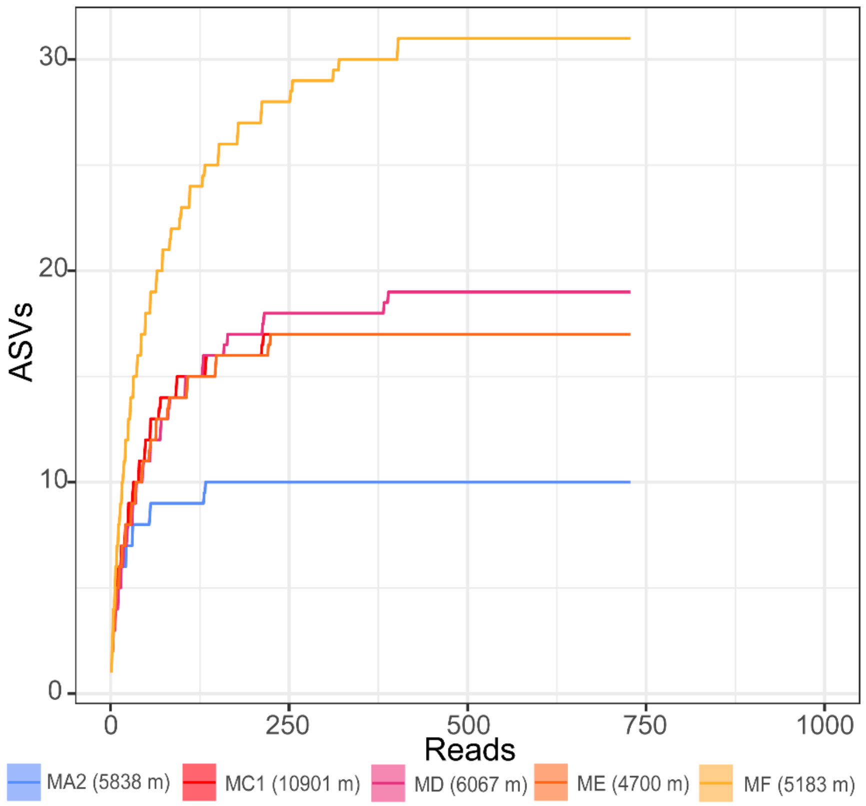

Figure S1. Rarefaction curves of fungal ASVs obtained from the analysis of sediment samples collected in the different benthic deep-sea sites investigated.

Table S1. Temperature and salinity values of the bottom waters and phytopigment, protein, carbohydrate, lipid and biopolymeric C concentrations in the surface sediments of the different deep-sea sites investigated. Mean values and standard deviations (±) are reported.

Site	Temperature (°C)	Salinity	Phytopigments (μg/g)	Proteins (mg/g)	Carbohydrates (mg/g)	Lipids (mg/g)	Biopolymeric C (mg/g)
MA2	1.572	34.686	1.65±0.475	0.26±0.004	1.10±0.047	0.22±0.063	0.73±0.068
MC-1	2.467	34.693	6.85±0.188	0.49±0.115	1.04±0.066	0.79±0.056	1.25±0.125
MD	1.595	34.686	2.50±0.141	0.41±0.021	1.25±0.095	0.18±0.018	0.84±0.060
ME	1.466	34.683	1.08±0.024	0.19±0.033	0.99±0.140	0.22±0.028	0.65±0.093
MF	1.501	34.685	1.21±0.011	0.35±0.052	1.17±0.040	0.26±0.027	0.84±0.060

Table S2. Outputs of the ANOVA main test (A) and pair pairwise comparisons test (B) carried out using the different trophic variables determined in the different benthic deep-sea sites investigated. CPE= Total phytopigments; PRT= proteins; CHO= carbohydrates; LIP= lipids; BPC= biopolymeric C.

A)					
Variable		df	Sum of squares	F	p-value
CPE	Site	4.0	69.528669	308.07626	0.0002
	Residual	10.0	0.564216		
PRT	Site	4.0	0.172435	12.146883	0.0007
	Residual	10.0	0.035490		
CHO	Site	4.0	0.128233	4.319594	0.0275
	Residual	10.0	0.074216		
LIP	Site	4.0	0.795605	109.390579	< 0.0001
	Residual	10.0	0.018183		
BPC	Site	4.0	0.640072	22.014705	< 0.0001
	Residual	10.0	0.072687		

B)					
	CPE	PRT	CHO	LIP	BPC
ME - MF	0.001	0.01	0.094	0.189	0.046
ME - MD	< 0.001	0.001	0.056	0.134	0.043
ME - MC-1	< 0.001	0.012	0.579	< 0.001	0.003
ME - MA2	0.108	0.022	0.257	0.983	0.302
MF - MD	< 0.001	0.158	0.27	0.019	0.943
MF - MC-1	< 0.001	0.144	0.045	< 0.001	0.007
MF - MA2	0.19	0.033	0.12	0.406	0.12
MD - MC-1	< 0.001	0.325	0.037	< 0.001	0.007
MD - MA2	0.041	< 0.001	0.074	0.398	0.109
MC-1 - MA2	< 0.001	0.026	0.28	< 0.001	0.003

Table S3. Outputs of the ANOVA main test (A) and pair pairwise comparisons test (B) carried out on fungal abundances (expressed as 18S rRNA copies g^{-1}) determined in the different benthic deep-sea sites investigated.

A)

	df	Sum of squares	F	p-value
Site	4	9.19E+16	32.321	< 0.0001
Residual	10	7.11E+15		

B)

	p-value
ME - MF	0.085
ME - MD	0.233
ME - MC-1	0.003
ME- MA2	0.2455
MF - MD	0.0508
MF - MC-1	0.0091
MF - MA2	0.0517
MD - MC-1	0.0026
MD - MA2	0.759
MC-1 - MA2	0.0026

Table S4. Variance inflation factor (VIF) for each covariate in the set of predictors selected.

Predictors	VIF
total phytopigment concentrations	2.408
carbohydrate concentrations	2.408

Table S5. Output of the best Poisson regression, resulting from a model selection, carried out using environmental variables (CPE, CHO) as predictors and 18S rRNA gene copies g^{-1} as response. CPE= total phytopigment concentrations; CHO = carbohydrate concentrations.

	Estimate	Std. Error	Pr(>|z|)
Intercept	1.645e+01	1.428e-04	<2e-16 ***
CPE	2.629e+00	9.411e-05	<2e-16 ***
CHO	3.637e-01	2.002e-04	<2e-16 ***
